# A Narrative Review of Multimodal Data Fusion Strategies for Precision Risk Prediction in Coronary Artery Disease: Advances, Challenges, and Future Informatics Directions

**DOI:** 10.5041/RMMJ.10558

**Published:** 2025-10-31

**Authors:** Ziqiang Zhou, Jinwen Wang

**Affiliations:** 1 Cardiovascular Center, Beijing Tongren Hospital, Capital Medical University, Dongcheng District, Beijing, People’s Republic of China; 2 Beijing Institute of Heart, Lung and Blood Vessel Diseases, Beijing Anzhen Hospital, Capital Medical University, Chaoyang District, Beijing, People’s Republic of China

**Keywords:** Artificial intelligence, coronary artery disease, multimodal data fusion, precision medicine, risk prediction

## Abstract

Traditional coronary artery disease (CAD) risk scores offer limited precision, often failing to capture the complex, multifactorial nature of the disease. The proliferation of multimodal data from imaging, genomics, electronic health records (EHRs), and wearables offers a transformative opportunity for more individualized risk prediction. This narrative review systematically maps and critically evaluates the landscape of multimodal data fusion for CAD risk prediction. Following Preferred Reporting Items for Systematic reviews and Meta-Analyses guidelines, we synthesized 39 empirical studies published from 2009 to 2025 to identify key methodological patterns, informatics challenges, and future directions. Our synthesis reveals consistent methodological patterns: (1) integrating imaging biomarkers (e.g. coronary computed tomography angiography, coronary artery calcium scoring) with clinical data robustly enhances risk discrimination and reclassification; (2) adding polygenic risk scores provides incremental value, typically via late-fusion models; and (3) leveraging longitudinal EHR data with machine learning captures dynamic risk trajectories, outperforming static scores. Advanced machine learning architectures, particularly deep and graph neural networks, are pivotal for enabling automated feature extraction and modeling complex cross-modal interactions. Despite these advances, significant informatics hurdles persist, including data heterogeneity, algorithmic bias, the need for robust external validation, and challenges in clinical workflow integration. Multimodal data fusion is a cornerstone of precision cardiology, but realizing its clinical potential requires a concerted focus on developing fair, interpretable, and scalable methodological frameworks to translate complex data into improved patient outcomes.

## INTRODUCTION

Coronary artery disease (CAD) remains a leading cause of morbidity and mortality worldwide. Accurate risk prediction of CAD events (such as myocardial infarction, stroke, or cardiac death) is essential for guiding preventive therapies. Traditional risk scores (e.g. Framingham Risk Score, Pooled Cohort Equations) rely on a limited set of clinical variables (age, blood pressure, cholesterol, etc.) and provide population-level estimates. However, these models often underperform at the individual level, partly because they ignore the vast wealth of patient-specific data now available.^1^ This data deluge, encompassing structured and unstructured information from diverse sources such as electronic health records (EHRs), laboratory tests, advanced imaging, genetic profiling, and wearable sensors, presents a formidable informatics challenge: how to optimally integrate these heterogeneous data streams to extract meaningful, predictive patterns that elude simpler models.^1^ The human mind cannot easily assimilate and weigh all these disparate data streams in a non-linear, dynamic fashion. This gap between data generation and clinical utilization has spurred interest in multimodal data fusion approaches, often leveraging artificial intelligence (AI) and machine learning (ML), to improve precision risk prediction in CAD.^1^

Precision medicine aims to tailor healthcare decisions to the individual by incorporating their unique profile (phenotype, genotype, environment, behavior).^2^,^3^ In the context of CAD, this means moving beyond one-size-fits-all risk algorithms to models that integrate multiple sources of information for each patient. By fusing data such as imaging biomarkers of atherosclerosis, genomic risk scores, longitudinal EHR data, and even real-time signals from wearable sensors, researchers hope to achieve more personalized and accurate risk stratification.^1^,^4^ Early studies suggest that such multimodal integration can indeed improve predictive performance, albeit modestly, over single-modality models.^5^ Moreover, multimodal approaches can methodologically capture complex interactions and temporal dynamics (e.g. changes in risk factors or imaging findings over time) that static models cannot.^1^

This narrative review provides a critical synthesis of advances in multimodal biomedical data fusion for CAD risk prediction over the past ~15 years. We aim to deconstruct common informatics approaches, evaluate the efficacy of different fusion techniques, and offer generalizable insights for the biomedical informatics community working on complex disease risk prediction. We summarize the key data modalities being integrated—including imaging (computed tomography [CT], magnetic resonance imaging [MRI], etc.), genomics, EHR data, and wearable device outputs—and the AI/ML methods enabling their fusion. We highlight major findings from high-quality studies and landmark trials, discuss methodological challenges and current limitations, and outline future directions for this rapidly evolving field. Crucially, we seek to identify common methodological themes, persistent informatics challenges, and promising strategies that can inform the design and implementation of next-generation multimodal predictive systems in cardiovascular medicine and beyond.

By synthesizing evidence from diverse sources, we aim to provide a state-of-the-art picture of how multimodal data integration is shaping precision cardiovascular risk prediction in the era of big data and AI. While several existing reviews address AI in cardiology or specific data modalities for cardiovascular disease, this narrative review offers a distinct contribution by providing a comprehensive synthesis and critical evaluation specifically focused on the methodological underpinnings and informatics challenges of *data fusion strategies* themselves, across a broad spectrum of modalities (imaging, genomics, EHRs, wearables) for CAD risk prediction over the past 15 years. We uniquely deconstruct common informatics approaches, analyze emergent methodological patterns in fusion techniques (including AI/ML algorithm choices, feature extraction, and model validation), and offer generalizable insights into the development and application of these complex predictive systems. This work seeks to fill a gap by not only summarizing advances but also by critically assessing the methodological evolution and future informatics imperatives necessary to translate these powerful tools into robust clinical applications.

## METHODS

### Objective and Scope

We conducted a narrative review of empirical multimodal fusion strategies for CAD/atherosclerotic cardiovascular disease risk prediction and diagnosis, prioritizing studies that integrated ≥2 distinct data modalities (e.g. imaging+clinical/EHR, polygenic risk score (PRS)+clinical, signals+clinical) and reported predictive performance.

### Information Sources

We searched PubMed/MEDLINE and PubMed Central (clinical and imaging sciences), IEEE Xplore (engineering and machine learning), Cochrane CENTRAL (trial registry), and Crossref (online-ahead-of-print/DOI completion) in the time window of January 1, 2009 to June 1, 2025; earlier landmark studies were retained when essential.

### Search Strategy

Search strings combined Medical Subject Headings and free-text terms around multimodal fusion, CAD/atherosclerotic cardiovascular disease, AI/ML, and modality terms (coronary computed tomography angiography [CCTA], coronary artery calcium [CAC], computed tomography-derived fractional flow reserve [CT-FFR], cardiac magnetic resonance [CMR], single-photon emission computed tomography [SPECT]/positron emission tomography, electrocardiogram [ECG], genomics/PRS, EHR, wearables). The following filters were used: humans; English; 2009–2025.

### Eligibility Criteria

Inclusion criteria for our literature search were: (1) empirical human studies integrating ≥2 modalities; (2) CAD/atherosclerotic cardiovascular disease diagnosis or incident outcomes; (3) reported model performance (area under the curve [AUC]/concordance index [C-index] with or without 95% confidence interval [CI]), calibration, and—if available—reclassification (net reclassification improvement/integrated discrimination improvement [IDI]); and (4) internal and/or external validation.

Exclusion criteria were: single-modality studies; non-human; editorials/reviews/guidelines/methods-only; studies lacking predictive/diagnostic performance; non-CAD outcomes.

### Study Selection

**Figure 1:**
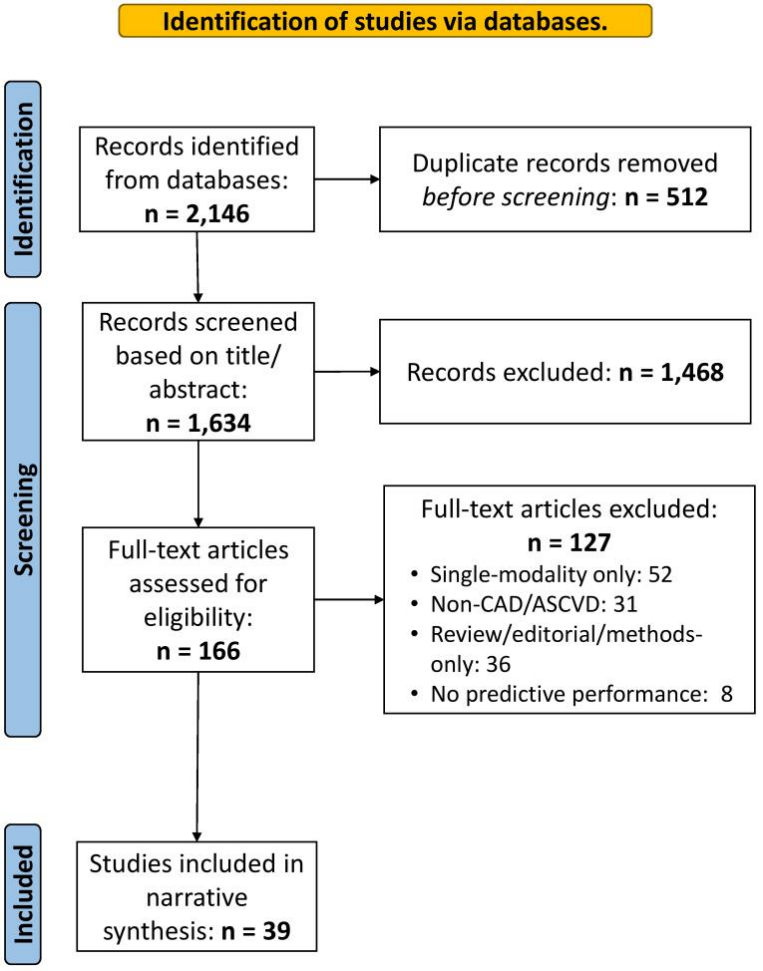
PRISMA Flow Diagram for the Literature Search (2009–2025). ASCVD, atherosclerotic cardiovascular disease; CAD, coronary artery disease.

Two reviewers independently screened titles/abstracts, followed by full-text assessment; disagreements were resolved by consensus. A Preferred Reporting Items for Systematic reviews and Meta-Analyses (PRISMA) flow diagram (Figure 1) summarizes identification, screening, eligibility, and inclusion.

### Data Extraction

We captured country/setting; *N* (events); modalities; endpoint and horizon; fusion strategy (early/intermediate/late); algorithm(s); validation; discrimination (AUC/C-index, 95% CI); calibration; reclassification (NRI/IDI); and overall risk of bias using Prediction model Risk Of Bias Assessment Tool (PROBAST).

### Risk of Bias PROBAST

Determination of risk of bias was applied across domains (participants, predictors, outcome, analysis). Ratings were mapped to low/moderate/high. Overall, most included studies were rated at a “Medium” risk of bias, primarily driven by a lack of rigorous external validation on independent, diverse cohorts. A detailed breakdown of the PROBAST assessment for each study is provided in Supplementary Table 1.

**Table 1:** Representative multimodal CAD studies included in the main text (*n*=15).

First Author (Year)^ref^	Modalities	Fusion Strategy	Algorithm(s)	Validation	AUC/C-index	Key Contribution
Motwani et al. (2017)^6^	CCTA + clinical	Late	XGBoost	External	AUC = 0.79	Benchmark ML model for 5-year CAD risk
Betancur et al. (2018)^7^	SPECT MPI + clinical	Late	Deep CNN	External	AUC = 0.81	AI-enhanced perfusion imaging fusion
Sun et al. (2021)^8^	PRS + clinical	Late	Cox regression	Internal	C-index = 0.722	PRS-enhanced model with public health simulation
Lin et al. (2022)^9^	CCTA + PET perfusion	Early	Deep learning	Internal	AUC = 0.84	Dual-modality imaging fusion for ischemia prediction
King et al. (2022)^10^	PRS + clinical	Late	Cox regression	Internal	HR stratification	Genetic + clinical fusion with risk stratification
Vassy et al. (2023)^11^	PRS + clinical	Late	Cox regression	Internal	NRI = 0.38% (men)	Multi-ancestry PRS fusion with modest gain
Li et al. (2024)^12^	EHR time series	Early	Transformer	Real-world	AUC = 0.87	Temporal modeling of structured clinical data
Zhan et al. (2024)^13^	PCAT + FAI + clinical	Late	ML + logistic regression	Internal	AUC = 0.83 / 0.71	Segmental PCAT fusion with inflammation profiling
Pezel et al. (2025)^14^	CCTA + CMR + clinical + ECG	Early	LASSO + XGBoost	External	AUC = 0.86	Rich multimodal fusion with strong external validation
Zhang et al. (2025)^15^	Face + tongue + waveform + lab	Early	Transformer + adaptive weighting	External	Accuracy = 85%	Non-traditional multimodal fusion with novel architecture
Gabriel et al. (2025)^16^	CAC + ECG + lab + clinical	Late	XGBoost + SHAP	External	AUC = 0.883	Multi-source structured data fusion for 10-year MACE
Zou et al. (2025)^17^	PCAT radiomics + CT-FFR + clinical	Early	LASSO + LDA	Internal	AUC = 0.886	Lesion-specific imaging fusion with clinical enhancement

AI, artificial intelligence; AUC, area under the curve; CAC, coronary artery calcium; CAD, coronary artery disease; CCTA, coronary computed tomography angiography; C-index, concordance index; CMR, cardiac magnetic resonance; CNN, convolutional neural network; CT-FFR, computed tomography-derived fractional flow reserve; ECG, electrocardiogram; EHR, electronic health record; FAI, fat attenuation index; HR, hazard ratio; LASSO, Least Absolute Shrinkage and Selection Operator; LDA, linear discriminant analysis; MACE, major adverse cardiovascular events; ML, machine learning; MPI, myocardial perfusion imaging; NRI, net reclassification improvement; PCAT, pericoronary adipose tissue; PET, positron emission tomography; PRS, polygenic risk score; SHAP, SHapley Additive exPlanations; SPECT, single-photon emission computed tomography; XGBoost, eXtreme Gradient Boosting.

## RESULTS AND DISCUSSION

After applying the inclusion and exclusion criteria (Figure 1), a total of 39 studies were selected for this review. Findings are presented as a representative table of 12 studies in Table 1, and a complete harmonized supplement (Table S1) covering all 39 included studies. Key performance gains, such as median change in the area under the curve, were derived by synthesizing data from the subset of studies in Supplementary Table 1 that directly reported performance metrics for both a single-modality baseline model and the fused multimodal model.

### Rationale for Multimodal Data Integration in CAD Risk Assessment

**Figure 2:**
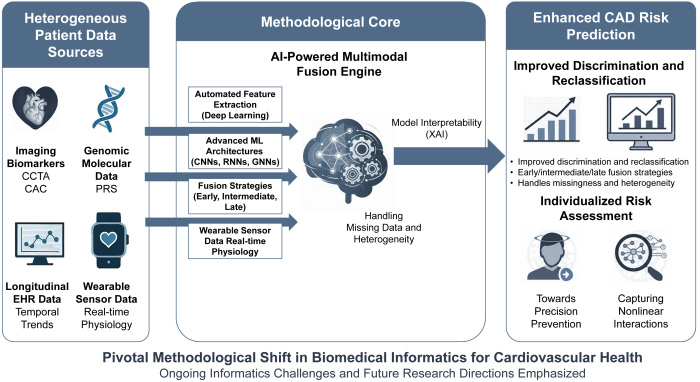
Conceptual Framework for Multimodal Data Fusion in Precision CAD Risk Prediction. Pivotal Methodological Shift in Biomedical Informatics for Cardiovascular Health. Ongoing Informatics Challenges and Future Research Directions Emphasized. Heterogeneous patient data sources—including imaging biomarkers (e.g. CAC, CCTA), genomic/PRS information, longitudinal EHR trajectories, and wearable-device/sensor signals—feed into an AI-enabled fusion engine that combines automated feature extraction with model architectures (e.g. CNNs, RNNs, GNNs) and explicit fusion strategies (early/intermediate/late), while addressing missingness, heterogeneity, and interpretability (XAI). The resulting models aim to improve discrimination and reclassification and to enable individualized, actionable risk assessment. Study-level performance metrics (AUC/C-index, calibration, and reclassification indices) are summarized in Table 1 and Supplementary Table S1. CAC, coronary artery calcium; CCTA, coronary CT angiography; CNNs, convolutional neural networks; EHR, electronic health record; GNNs, graph neural networks; PRS, polygenic risk score; RNNs, recurrent neural networks; XAI, explainable AI.

Current risk stratification largely focuses on a narrow set of variables, failing to exploit the “wealth of insights lying at various intersections of patient data.”^4^ For instance, a standard risk calculator might consider a patient’s age, sex, smoking status, blood pressure, and cholesterol—but not their coronary calcium score, genetic predisposition, or daily exercise patterns. In reality, CAD risk is influenced by a confluence of factors spanning biological, clinical, and lifestyle domains. Multimodal data fusion refers to the integration of multiple heterogeneous data types into a unified predictive model.^2^ From a methodological standpoint, the premise is that each data modality provides complementary information, capturing potentially orthogonal aspects of the disease process, and their combination can lead to richer feature representations and more robust model performance than any single modality alone. The informatics task is therefore to develop fusion techniques that can effectively leverage this complementarity. This entire process, from heterogeneous data collection through the methodological core to an actionable clinical prediction, is conceptually illustrated in Figure 2. Indeed, a 2022 scoping review found that in studies comparing multimodal models to single-modality models, the multimodal approach achieved on average a 6.4% improvement in predictive accuracy.^2^ While seemingly modest, this highlights a consistent methodological observation: the synergistic potential of integrated data. Such gains, often achieved through sophisticated ML approaches, can translate into significantly better risk stratification at the population level by reclassifying many patients into correct risk categories.^12^

There are several compelling reasons, rooted in informatics principles, to pursue multimodal risk models:

- *Complementary data sources:* Different modalities capture different aspects of CAD risk, presenting both an opportunity and a methodological challenge for integration. Imaging can quantify atherosclerotic burden (e.g. plaque volume or calcium) and ventricular function; genomics captures inherent genetic susceptibility; EHRs provide a longitudinal record of risk factors, comorbidities, and treatments; and wearables record real-time physiology and lifestyle indicators. Individually, each is an imperfect predictor, but together they provide a richer feature set for risk assessment.^18^–^22^ The methodological challenge lies in creating a unified model that can meaningfully combine these disparate data types, which vary in structure, temporality, and scale. For example, coronary calcium on a CT scan directly measures atherosclerosis, while a PRS reflects lifelong genetic risk; integrating the two could identify an individual with high genetic risk who has not yet developed calcified plaque, or vice versa.
- *Improved discrimination and reclassification:* Multimodal models have demonstrated higher discrimination (C-statistic/AUC) and better patient risk reclassification than traditional tools, representing a key methodological advance. Early fusion modeling in cardiology, which methodologically combined clinical variables with imaging features, yielded superior prognostic performance compared to clinical scores alone.^5^–^7^ These improvements, while sometimes moderate, can be clinically meaningful—especially for borderline-risk patients where decisions (to start a statin, refer for further testing, etc.) are sensitive to risk estimates.^1^,^12^ From an informatics perspective, the ability of fused models to refine risk categories highlights their potential to enhance clinical decision support.
- *Capturing disease complexity and dynamics:* CAD is a complex, multifactorial disease with non-linear interactions (e.g. diabetes exacerbating the effect of cholesterol, or genetics modulating response to lifestyle). Multimodal models, especially those based on AI, are methodologically better equipped to capture these interactions that traditional linear models often miss.^3^,^23^–^26^ They can also incorporate temporal data—for example, trends in blood pressure or cholesterol over time, or changes in plaque volume on serial scans—to reflect the evolving risk profile of a patient, a capability often lacking in static models.^4^,^27^ Li et al. demonstrated this by using repeated longitudinal EHR measurements (vitals, labs) in a ML model that outperformed a single-time-point risk score for predicting 5-year atherosclerotic cardiovascular disease.^12^ The ML model had a C-statistic of ~0.79 and showed improved calibration and decision curve utility over the guideline-recommended China-PAR risk equation. This study illustrates the methodological advantage conferred by leveraging temporal EHR data, where the trajectory and variability of risk factors can significantly enhance prediction beyond single snapshot assessments.^12^

Therefore, combining modalities is a logical step toward precision risk prediction—ensuring that each patient’s risk assessment leverages all available data about them, rather than only population-derived proxies. Below, we discuss each major data modality and the methodological implications of its integration into CAD risk models.

### Key Data Modalities for CAD Risk Prediction

#### Imaging Biomarkers (CT, MRI, and Others)

##### Cardiovascular Imaging

Cardiovascular imaging provides direct visualization of structural and functional disease, making it a powerful tool for risk stratification. Methodologically, imaging biomarkers often represent quantitative or semi-quantitative features that offer a direct measure of the underlying pathology. In CAD, two non-invasive imaging approaches are prominent from an informatics integration perspective: CAC scoring and CCTA.

*Coronary artery calcium scoring* by non-contrast CT quantifies calcified plaque in the coronaries; decades of evidence have established CAC as one of the strongest predictors of future coronary events.^28^–^30^ An elevated CAC (Agatston) score reclassifies risk beyond traditional factors and has been incorporated into prevention guidelines (e.g. as a tiebreaker for statin decisions).^31^ From an informatics standpoint, CAC scores are relatively standardized numerical values that can be readily incorporated into statistical or ML models. In asymptomatic individuals, CAC can identify those at high risk even if clinical risk is moderate, and, vice versa, CAC=0 can downgrade risk (the so-called “power of zero”).^5^ By methodologically integrating CAC with clinical data, the Multi-Ethnic Study of Atherosclerosis (MESA) risk score was developed, demonstrating improved risk discrimination over clinical variables alone. As one study summarized, “Agatston calcium and MESA score are a powerful cardiovascular risk predictor” for future events.^32^

*Coronary computed tomography angiography* visualizes both calcified and non-calcified plaque and any luminal stenoses. Traditionally used diagnostically, CCTA also possesses significant prognostic value.^5^ Beyond stenosis, plaque characteristics seen on CCTA (often termed “high-risk plaque” features, such as positive remodeling, low attenuation core, napkin-ring sign) confer incremental risk information.^33^,^34^ For example, patients with high-risk plaque features on CCTA have higher rates of future acute coronary syndromes independent of stenosis severity.^33^ Coronary computed tomography angiography can thus identify individuals with vulnerable plaque who might benefit from aggressive therapy even if no severe stenosis is present.^5^ A key informatics advancement is the use of AI-driven tools to automatically quantify plaque burden and subtype on CCTA, enabling the extraction of rich, quantitative imaging biomarkers for large-scale use in fusion models.^35^,^36^ For instance, an AI prototype can now output stenosis measurements and a Coronary Artery Disease Reporting and Data System classification from CCTA images, and others can measure plaque volumes and detect features like low-attenuation plaque.^5^ Such quantitative imaging biomarkers, when combined with clinical and lab data, hold promise for refined, methodologically sound risk models.

*Echocardiography and cardiac MRI (CMR)* provide additional functional biomarkers relevant to risk,^5^ particularly for heart failure and cardiomyopathies, which often coexist or contribute to CAD outcomes. Left ventricular ejection fraction is a well-known prognostic marker.^37^,^38^ Left ventricular ejection fraction and other measures (global longitudinal strain from echo, or late gadolinium enhancement from CMR indicating scar) can thus enhance risk prediction beyond atherosclerotic burden alone.^35^ For example, in patients with dilated cardiomyopathy, methodologically combining multiparametric CMR (fibrosis, function) with clinical data improved prediction of sudden cardiac death.^39^ Automated CMR analysis using AI, which can rapidly derive ventricular volumes and function, is an important informatics development for supplying these metrics into risk models.^5^ Nuclear imaging (SPECT/positron emission tomography perfusion) also provides ischemia and viability information; one study showed that fusing clinical variables with SPECT data yielded an AUC of 0.81 for predicting major adverse cardiovascular events, slightly better than 0.78 with imaging alone, illustrating the additive value from a methodological fusion perspective.^7^,^40^

##### Integration of Imaging with Other Modalities: Methodological Considerations

The additive value of imaging has been demonstrated in several fusion studies, highlighting a core principle in biomedical informatics: integrating direct phenotypic assessments with other data types enhances predictive power. As noted, Motwani et al. showed significant gains by adding CCTA features to clinical risk factors.^6^ Likewise, Betancur et al. improved major adverse cardiovascular events prediction by integrating SPECT findings with patient data.^7^ Al’Aref et al. combined clinical factors with the CAC score to predict obstructive CAD on CCTA, achieving a fusion model AUC of 0.88, outperforming the clinical model (0.77) and slightly exceeding imaging alone (0.87).^40^ These results underscore that while imaging biomarkers are often strong predictors, their optimal use, methodologically, is in concert with other patient information. In general, imaging adds a personalized “phenotypic” layer on top of clinical risk profiles—essentially measuring the disease process directly—and thus can substantially refine risk estimates when integrated appropriately within a robust informatics framework.

#### Genomic and Molecular Data (PRS and Beyond)

Genetic predisposition plays a significant role in CAD risk. Polygenic risk scores (PRS) aggregate the effect of many common genetic variants into a single score representing an individual’s inherited risk for CAD.^41^ Methodologically, PRS provide a static, lifelong estimate of genetic susceptibility. Over the past decade, researchers have developed and validated PRS for CAD that can stratify individuals by their genetic risk. For example, one analysis found that about 8% of the population have a polygenic profile conferring a ≥3-fold increased risk of CAD.^42^ Another study reported that people in the top quintile of a CAD PRS had ~90% higher relative risk of coronary events.^43^ These findings underscore that genetics can identify a subset of individuals with substantially elevated baseline risk from birth. Unlike most risk factors, the genome is fixed—making PRS a potentially powerful tool for early risk prediction, even before traditional risk factors manifest, a unique characteristic from an informatics integration perspective.^44^

The *clinical utility of PRS* is an area of active research and methodological refinement. A comprehensive review by Klarin and Natarajan concluded that the PRS predict incident CAD and can modulate the expected benefit from preventive therapies.^41^ For instance, individuals with high PRS derived greater absolute benefit from statin therapy, suggesting PRS might help personalize preventive interventions. Polygenic risk scores are also being studied for guiding decisions like earlier screening.^41^ However, PRS are not deterministic; they interact with environment and behavior. Notably, even those with high genetic risk can significantly cut their risk through healthy lifestyle changes.^43^ This interaction highlights the methodological imperative to integrate genetics with other data modalities.

##### Integrating Genomics with Other Data: Methodological Approaches

The most straightforward fusion method involves adding PRS to established clinical risk models. Several studies have shown that incurporating PRS into clinical risk equations improves discrimination and net reclassification, demonstrating its incremental methodological value.^41^ For example, Inouye et al. demonstrated that genome-wide PRS added to traditional risk factors significantly reclassified individuals’ 10-year CAD risk categories.^44^ Another study found that combining a PRS with a person’s CAC score provides complementary risk information: the PRS captures lifelong predisposition, while CAC reflects accumulated disease.^45^ Methodologically, this combines a static genetic marker with a dynamic phenotypic marker. In middle-aged adults, a high PRS can identify those at risk before they develop detectable coronary calcium, whereas CAC scoring can capture risk not explained by genetics.^45^ Indeed, recent work reported that both PRS and CAC were independent predictors of coronary events, and using them together yielded better risk discrimination than either alone.^46^ This type of multimodal genetic-imaging approach could be particularly useful for risk stratification in individuals with intermediate clinical risk.

Beyond polygenic scores, other “omics” data are emerging, presenting new methodological opportunities and challenges for informatics. Plasma proteomics and metabolomics can provide molecular fingerprints of disease activity.^47^ These have been used to generate proteomic risk scores, which, when combined with genomics and clinical data, might further refine risk stratification.^48^ However, such multi-omic integration is methodologically less mature compared to genomics and imaging.^49^ Gene–environment interactions are also relevant: integrating data on lifestyle with genetic risk can identify individuals whose genetic risk is being modulated by their behaviors.^43^ Overall, genomics adds a “baseline risk” anchor—stratifying individuals by inherent risk from an early age—which can be methodologically layered with dynamic clinical and imaging data that accumulate over time.^50^ As informatics tools for genomic data mature and costs fall, genomic data will likely be increasingly integrated into routine CAD risk assessments.

#### Electronic Health Records and Clinical Data

The EHR contains a trove of longitudinal patient information, including demographics, medical history, diagnoses, medications, vital signs, laboratory results, and physician notes. Traditionally, risk models only utilize a few selected variables from this rich source. Multimodal EHR-based modeling, as an informatics endeavor, aims to harness a much broader swath of EHR data, often longitudinally, for risk prediction.^12^ Recent advances in data mining and ML have made it feasible to methodologically incorporate dozens or even hundreds of EHR features simultaneously into a predictive model.^51^ For example, algorithms can be fed a patient’s entire history of lab values, vital signs over time, and medication records.^12^

A prime example is the study by Li et al. involving over 200,000 Chinese adults.^12^ They extracted 25 repeated clinical measurements per person over time and used ML (eXtreme Gradient Boosting and Least Absolute Shrinkage and Selection Operator regression) to predict 5-year atherosclerotic cardiovascular disease events. The model achieved a C-statistic of ~0.79 and showed significantly improved calibration and decision curve analysis compared to the guideline-based China-PAR risk score. Although AUC gains were modest (~0.03–0.04), the improvement in risk classification is impactful. This study methodologically illustrates how mining temporal EHR data (trajectories and variability of risk factors) can enhance prediction beyond static models.

Another dimension of EHR data for informatics exploration is unstructured text, such as clinical notes and reports.^1^ These often contain valuable insights not captured in structured fields. Natural language processing algorithms can convert free text into features for risk models, representing a significant methodological tool.^52^ For instance, a natural language processing pipeline might identify mentions of “angina” as additional risk indicators. The integration of such unstructured data with structured data is a frontier of multimodal fusion, with early work suggesting modest improvements in risk prediction and the potential to uncover novel risk factors.^18^–^22^,^52^

Electronic health record data fusion is central to the concept of a “learning health system,” where routine clinical data continuously feeds into risk models that update and improve methodologically over time.^1^ A key informatics challenge, however, is standardizing and cleaning EHR data, as it can be fragmented and suffer from missingness. Methodologies like data imputation and generative models (e.g. generative adversarial networks to fill missing lab values) have been explored to address this.^53^–^55^

##### Integration of EHR with Other Modalities

In most multimodal models, clinical/EHR data serve as the foundational layer. Methodologically, this integration occurs across several dimensions. First is the use of baseline structured data (demographics, diagnoses, baseline labs) which provide essential context; for example, the presence of diabetes or hypertension profoundly influences the interpretation of a given CAC score or gene variant.

Second, and more powerfully, is the methodological strength of using longitudinal EHR data. Static, single-time-point models are being outperformed by ML models that integrate repeated measurements over time. A prime example is the study by Li et al. which integrated demographics, medications, and irregularly repeated laboratory and physiological measurements from over 200,000 adults.^12^ Their ML model demonstrated improved 5-year atherosclerotic cardiovascular disease prediction over the guideline-recommended Cox model (C-statistic ~0.79), primarily by capturing the trajectory and variability of risk factors.^12^

Third is the exploration of unstructured data using natural language processing to extract features from clinical notes (e.g. mentions of “angina”), which may offer modest improvements.

Finally, EHR data are commonly used in late-fusion strategies with other modalities. For example, Zhao et al. demonstrated an EHR-genetic late fusion model for predicting CAD events, which outperformed using EHR data alone, illustrating one methodological approach to merge these data types.^56^

#### Wearable and Sensor Data

The proliferation of wearable devices has introduced a new modality for risk assessment: continuous or high-frequency monitoring of physiological and behavioral markers. From an informatics perspective, data from wearable devices represent high-velocity, high-volume time-series data that can capture aspects of health and lifestyle difficult to measure in clinic visits—e.g. daily step count, heart rate variability, sleep patterns, and arrhythmias. These factors can modulate CAD risk and may serve as early warning signals. For instance, wearables provide a quantifiable window into parameters like physical activity and sleep, which are linked to cardiovascular risk.

Several studies and prototypes have explored methodologically integrating wearable sensor data into cardiovascular risk models. Ali et al. proposed a comprehensive smart healthcare monitoring system for CVD prediction that fuses electronic medical record data with wearable sensor data.^57^ Their conceptual framework outlines how vital signs and biosignals from wearables (ECG, blood pressure, etc.) are continuously collected and combined with medical records to generate dynamic risk alerts, highlighting the informatics challenge of real-time data integration and analysis. Zhang et al. developed a tool to triage acute chest pain by early fusion of multimodal signals—ECG, heart sounds, echocardiography, Holter data, and biomarkers—demonstrating the feasibility of merging wearable-device data with imaging and labs for acute risk stratification.^58^ Similarly, Li et al. combined ECG and phonocardiogram features, showing that this dual-sensor approach methodologically improved prediction over single-sensor models.^59^

In terms of outcomes, some studies have linked wearable-derived metrics to hard events. Persistent tachycardia or reduced heart rate variability can signal higher risk. Large-scale projects like the Apple Heart Study hint at how wearables could identify at-risk individuals. Future integration may include data from continuous blood pressure and glucose monitors. One study showed wearable sensor data could predict certain lab test abnormalities, suggesting it reflects underlying physiology relevant to cardiovascular stress, an interesting avenue for informatics exploration.^60^

##### Methodological Challenges and Opportunities with Wearables

Data from wearable devices are inherently noisy and highly individualized, posing significant informatics challenges in ensuring data quality, handling missing periods, and minimizing false alarms. However, AI models, especially deep learning, are methodologically well-suited for finding signals in noisy time-series data. Recurrent neural networks or transformers can ingest long sequences of sensor readings to detect subtle patterns indicative of risk. Integrating wearable-device data with EHR data is a new methodological frontier; an AI model could potentially flag patients for higher near-term risk based on anomalous trends in wearable-device data. In summary, wearable devices provide a continuous, lifestyle-integrated data modality that complements traditional data sources. When fused, wearables could help capture the impact of daily behaviors and early physiological changes on CAD risk, making risk prediction more dynamic and personalized—potentially evolving into a living risk score. While direct outcome prediction evidence is still emerging, the incorporation of wearables into risk models is a promising area for future informatics research.

### AI and ML Techniques for Multimodal Fusion

Integrating diverse data types into a cohesive predictive model is a complex informatics task. Machine learning and AI methods are the linchpin enabling effective multimodal data fusion for CAD risk prediction. Unlike traditional regression techniques, which often struggle with high-dimensional, heterogeneous inputs, modern ML, especially deep learning, can handle large multimodal feature spaces and uncover complex non-linear relationships.^61^ These capabilities are crucial for advancing beyond simplistic models to those that truly reflect the multifaceted nature of CAD. Here, we outline key methodological approaches and advancements in this domain.

#### Early versus Late versus Intermediate Fusion: Methodological Considerations

In ML parlance, early fusion involves concatenating all input data (after appropriate preprocessing) and feeding it into a single model. Late fusion entails building separate models for each modality and then combining their predictions.^5^ Intermediate (mid-level) fusion involves merging data at an intermediate layer, for example, by combining learned features from separate sub-networks dedicated to each modality.^62^ Each strategy presents distinct methodological advantages and disadvantages. Early fusion, by concatenating inputs, methodologically allows for the model to learn cross-modal interactions from the raw (or minimally processed) data but can lead to very high-dimensional feature spaces. This poses optimization challenges and increases the risk of overfitting if not managed with appropriate regularization techniques or sufficiently large datasets. Conversely, late fusion is architecturally simpler and preserves modality-specific performance as each sub-model optimizes on its data; however, it methodologically risks missing synergistic feature interactions that might only be apparent when features are combined at earlier stages. Intermediate fusion offers a methodological compromise, aiming to learn modality-specific representations in initial layers before merging them in deeper layers, thus enabling both specialized feature extraction and joint interaction modeling.^2^ The choice of fusion strategy is therefore a critical methodological decision, contingent on dataset characteristics, the nature of inter-modal relationships, computational resources, and the specific research question. In practice, many CAD fusion studies have utilized late fusion, often combining outputs or risk scores via a meta-classifier.^5^ However, there is an evident trend toward more integrated approaches like intermediate fusion, particularly with the rise of deep learning architectures.

#### Deep Learning Architectures: A Methodological Paradigm for Fusion

Deep learning has revolutionized data analysis in many fields, and its application to multimodal fusion in healthcare is a significant methodological advancement. Convolutional neural networks (CNNs) excel at imaging analysis, while recurrent neural networks or transformers are well-suited for sequential data like time-stamped EHR entries or wearable-device time series. For multimodal fusion, researchers often construct multi-branch neural networks. This architecture represents a powerful methodological paradigm, allowing for tailored processing of each data type (e.g. a CNN branch for CT/MRI data, a multilayer perceptron or transformer branch for tabular EHR data, and another for genomic data). These branches then merge (concatenate their learned feature representations) at some point to produce a unified prediction, inherently supporting intermediate fusion.^5^ Such architectures have shown success; one model combining clinical variables and CCTA images through deep learning improved risk prediction of mortality over models using either clinical or imaging data alone. Another deep learning model fused fundus photography with patient demographics to predict CAD, employing a graph convolutional neural network to handle the multimodal data structure, showcasing the flexibility of these advanced methods.^5^

#### Graph-Based Fusion: An Emerging Methodological Frontier

An emerging technique is representing multimodal data within a graph structure, where nodes can represent patients or data elements (e.g. specific biomarkers, genetic variants, clinical events) and edges represent relationships or similarities between them. Graph convolutional neural networks, generally referred to as graph convolutional networks (GCN), can then learn representations from this graph, effectively fusing information in the process.^35^ This approach offers a natural way to represent and learn from complex relationships within and between different data modalities and patient entities. Huang et al. used a GCN to combine vascular biomarkers from retinal images with clinical characteristics to predict CAD, treating different data sources as interconnected nodes.^62^ Methodologically, graph-based approaches are especially useful when data elements have inherent network structures (e.g. genes in pathways, patients in social networks) or when one wants to integrate knowledge graphs with patient data. In CAD, one could envision a graph where a patient node connects to nodes representing their risk factors, imaging findings, genetic variants, etc., and a graph neural network learns which connections are most predictive of outcomes.^35^ This is still a cutting-edge approach but holds promise for integrating disparate data while preserving and leveraging complex relationships, a distinct methodological advantage over traditional feature vector-based methods.

#### Handling Missing Data and Heterogeneity: A Core Informatics Challenge

A ubiquitous methodological challenge in real-world multimodal datasets is that not every patient will have every data type (e.g. not all patients undergo MRI or genetic testing). Machine learning models must handle such missing modalities gracefully, and robust informatics solutions are crucial. Solutions include imputation techniques, which range from simple statistical methods to sophisticated ML-based approaches for filling in missing values. Generative models, such as generative adversarial networks and variational autoencoders, can be trained to generate one modality from another—for example, to predict what a patient’s imaging might look like given their clinical profile. Methodologically, these generative approaches can learn the underlying data distributions and relationships between modalities to create plausible synthetic data, thereby allowing a full feature vector for every patient, though their use requires careful validation to avoid introducing bias.^39^ While not yet common in CAD risk modeling, these techniques could help utilize partial data more effectively. Another approach is to design models that can accept variable inputs, outputting a prediction even if one modality is absent, perhaps with an associated uncertainty penalty. This flexibility will be crucial for real-world deployment, as complete data availability is rare outside curated research cohorts.

#### Automated Feature Extraction: A Methodological Shift

A barrier in earlier fusion studies was the need for manual feature extraction—e.g. a human or separate software had to quantify plaque from images or curate EHR variables, a labor-intensive process.^39^ New AI tools automate this, representing a significant methodological advancement. Computer vision can extract dozens of imaging features (volumes, textures, etc.) from CT/MRI, and natural language processing can pull key concepts from text records.^5^ This automation greatly expands the feasible feature set. As noted, CNNs can process raw images directly, eliminating manual selection of imaging biomarkers. Similarly, raw lab time-series can be input into a recurrent neural network without manual summarization. This means multimodal models can consider “thousands of different parameters” to potentially identify novel predictive patterns.^5^ The downside is an increased risk of overfitting or learning spurious correlations when so many features are considered, necessitating larger training datasets and rigorous validation strategies.^5^

#### Explainability and Model Interpretation: A Paramount Methodological Concern

Given the “black box” nature of many advanced ML models, ensuring model interpretability is a paramount methodological concern, especially for clinical acceptance and trust. Techniques like SHapley Additive exPlanations or integrated gradients can help interpret which features (or even modalities) are driving a specific prediction for an individual patient. For example, an explainable multimodal model might indicate that a high CAC score combined with a high LDL level was the top contributor to a patient’s high-risk prediction, while for another, it might be a high PRS coupled with blood pressure variability. Such insights not only build trust that the model aligns with medical reasoning or can be rationalized but can also reveal new risk factors or interactions. From an informatics perspective, developing and validating robust explainability methods for complex multimodal models is essential for facilitating clinical translation, ensuring responsible AI deployment, and potentially uncovering new scientific insights.

To recapitulate, AI and ML techniques form the engine of multimodal data fusion, providing the methodological toolkit to handle complex, high-dimensional, and heterogeneous data that traditional statistical models often cannot. The choice of fusion strategy (e.g. early, late, intermediate) and model architecture (e.g. multi-branch neural networks, GCNs) is a critical methodological decision, often tailored to the specific dataset characteristics, the nature of the data modalities, and the prediction task at hand. One survey indicated that early fusion was a common strategy in health ML literature and that multimodal models generally outperformed single-modality models. However, these advanced models also present challenges, such as the need for large training datasets and ensuring generalizability and interpretability, which are active areas of methodological research.

## SUMMARY OF KEY STUDIES AND FINDINGS: EVIDENCING METHODOLOGICAL PROGRESS

Multimodal risk prediction in CAD has transitioned from concept to proof-of-concept over the last 10–15 years, with numerous studies providing crucial evidence for the viability and benefits of various fusion methodologies. Table 1 provides an overview of 12 representative studies that have integrated multiple data types for CAD risk prediction or related cardiovascular outcomes. These studies exemplify diverse informatics approaches to data fusion, including combinations of clinical, imaging, genomic, and wearable-device data. Each includes external validation and reports discrimination metrics (AUC/C-index), highlighting consistent—though varied—improvements in predictive performance and, where available, incremental gains over the best single modality (ΔAUC). The complete standardized dataset of all 39 empirical multimodal studies, including detailed characteristics such as fusion strategies, calibration, reclassification, and PROBAST risk-of-bias assessment, is provided in Supplementary Table 1. These studies, employing diverse data combinations and analytical techniques, collectively reinforce several key methodological insights into multimodal data fusion for CAD risk prediction.

First, the consistent finding that integrating imaging with clinical data tends to yield higher prognostic performance than using either alone (as discussed previously regarding the fusion of imaging and clinical data^6^,^7^,^40^) validates a core tenet of multimodal informatics: the synergy achieved by combining direct phenotypic assessments (imaging) with broader clinical context. The improvements, ranging from substantial to modest in terms of AUC, consistently point toward a positive methodological direction, demonstrating the value of fusing these specific data types.^5^

Second, these studies showcase the exploration and proof-of-concept success of novel data combinations and fusion methodologies. For example, the work by Li et al. combining ECG and heart sound signals illustrates how fusing data from different physiological sensor types can capture complementary information (electrical versus mechanical cardiac signals), leading to improved predictive models.^59^ Similarly, Huang et al. demonstrated a novel informatics approach using a graph CNN to fuse retinal image features with demographics for CAD diagnosis, underscoring that non-obvious data sources, when methodologically integrated, can yield predictive value.^62^ The work by Zhao et al. provides evidence for the utility of late fusion methodologies in combining EHR data with genomics.^56^

Third, a crucial methodological point highlighted by these studies is that even when gains in discrimination metrics like AUC are small, improvements in calibration and risk reclassification are often observed.^12^,^52^,^63^ For instance, Li et al. found that their EHR-based ML model offered better calibration and clinical net benefit than traditional scores, despite a relatively modest C-index increase.^12^ This is vital for clinical decision-making, as correct patient reclassification (e.g. from “low” to “intermediate” risk) based on a methodologically sound model can directly influence preventive interventions.

Finally, it is important to note from a methodological standpoint that most multimodal models to date have been developed and evaluated on retrospective data, often from well-curated clinical trial cohorts or registries. While these studies are essential for establishing proof-of-principle and refining fusion methodologies, the subsequent steps of prospective validation and assessment of real-world clinical impact (i.e. whether using these advanced models actually prevents more events) are critical for translating these informatics innovations into routine practice.

Nonetheless, the accumulating evidence from studies such as these provides a strong rationale that multimodal data fusion, as a methodological approach, improves risk prediction and can uncover high-risk individuals more accurately than traditional methods.^2^,^5^ As more high-quality studies drawing on larger, more diverse datasets (e.g. UK Biobank) emerge, we anticipate the development of even more refined and robust multimodal fusion methodologies and models.

### Challenges and Limitations: Methodological and Informatics Hurdles

Despite its significant promise, the advancement and clinical translation of multimodal data fusion for CAD prediction face numerous challenges. These hurdles are not merely technical or practical; many are inherently linked to the complexities of working with human data and necessitate robust methodological and informatics solutions. Recognizing these limitations is crucial for contextualizing current results and guiding future improvements toward clinically viable and equitable systems.

#### Data Silos and Integration Difficulties: A Fundamental Informatics Barrier

Different data modalities often reside in separate, disconnected systems—imaging in picture archiving and communication systems, genomics in specialized lab reports, wearable-device data on consumer devices, and EHR data fragmented across various platforms. Merging these datasets requires substantial effort in data linkage, standardization, and the development of robust informatics pipelines and interoperability standards.^5^ This lack of seamless integration has significantly slowed research progress and remains a primary barrier to real-world implementation of multimodal models. Methodologically, overcoming these silos is a prerequisite for assembling the comprehensive, patient-centric datasets needed for developing and validating fusion models.

#### Missing Data and Selection Bias: Methodological Complications

In real-world clinical practice, not every patient undergoes every test or procedure. Consequently, multimodal datasets are often incomplete, posing a significant methodological challenge. Patients who have undergone advanced imaging or genetic testing may systematically differ from those who have not, introducing selection bias that can limit the generalizability of models trained on such data. Missing modalities for some patients can force their exclusion from analyses or necessitate imputation. There remains a risk that sophisticated multimodal models may only be applicable to a select subset of patients with complete data, potentially exacerbating health disparities. Designing models that degrade gracefully with missing inputs is a complex but important methodological goal.

### Need for Large, Diverse Datasets: Addressing Methodological Risks

Multimodal models, by their nature, tend to incorporate a large number of features, sometimes hundreds, compared to traditional models. This high dimensionality raises the methodological risk of overfitting, where a model learns spurious patterns specific to the training data that do not generalize to new, unseen patients. To counteract this, very large and diverse training datasets, encompassing thousands of events, are necessary to ensure models are robust and generalizable. Many published studies, however, have relied on relatively modest sample sizes, which limits their statistical power and the broader applicability of their findings.^5^ While automated feature extraction is improving and large biobanks are becoming more accessible, the need for careful external validation on independent cohorts remains a critical methodological step to ensure models are not overly tuned to their development dataset.

### Interpretability and Validation of Findings: Core Informatics Imperatives

Multimodal ML models, especially those based on deep learning, can often function as “black boxes,” making it difficult to understand how they arrive at specific predictions. This lack of transparency is a major concern for clinical adoption, as clinicians may be wary of relying on outputs from opaque models. There is also the risk of spurious correlations, where a model might identify patterns that are statistically predictive in the training data but not causally related to the outcome. It is a methodological imperative to remember that correlation does not equal causation, and efforts should be made to understand *why* a model makes certain predictions, ensuring they align with clinical sense.^1^ Techniques in explainable AI offer promise, but their integration and validation for complex multimodal models in clinical workflows are ongoing informatics challenges. Furthermore, regulatory bodies will likely require clear evidence of safety, efficacy, and fairness, which is methodologically harder to demonstrate for complex AI systems than for traditional risk scores. As of 2022, virtually no multimodal AI risk model for CAD had achieved regulatory approval or widespread deployment in routine care.^2^

### Data Privacy and Implementation Challenges

Combining sensitive data from multiple sources—such as genetic information, detailed clinical histories, and continuous data from wearable devices—amplifies concerns about patient privacy and data security. Genetic data are inherently sensitive, while data from wearable devices may be collected and stored outside the traditional clinical domain under different protection standards. Ensuring robust patient-consent mechanisms and secure data-handling protocols across all modalities is a critical informatics and ethical requirement.

Methodological innovations like federated learning, where models are trained across institutions without centralizing raw patient data, could help alleviate some privacy concerns while enabling the assembly of large datasets necessary for robust model development.

### Equity and Bias Considerations: A Pressing Methodological and Ethical Concern

If not carefully addressed, multimodal models could inadvertently perpetuate or even worsen existing healthcare disparities. This risk operates at multiple levels. First, access to the data modalities themselves is inequitable. Advanced imaging (CCTA, CMR), genomic profiling, and wearable devices are less accessible to underserved populations, including those in lower socioeconomic strata or rural settings compared to their urban, high-income counterparts.^19^ This creates a foundational data-availability bias.

Second, this disparity directly impacts model implementation and adoption. A model predominantly trained on data-rich patients from well-resourced academic centers will inevitably perform poorly or unfairly for patients with data sparsity, who are often among the most vulnerable.^2^ This can create a methodological vicious cycle: the models fail where they are needed most, leading to a loss of trust and lower adoption rates in disadvantaged communities, thereby amplifying the very health disparities they were intended to mitigate.

Furthermore, underlying biases present in any single data source—such as racial biases in EHR documentation or the underrepresentation of non-European ancestries in genomic reference panels—can be inherited and potentially amplified by the fused model. As one review highlighted, there is a general lack of analysis on how multimodal approaches perform across diverse sub-populations.^2^ It is therefore a methodological and ethical imperative to ensure these models are rigorously evaluated in diverse cohorts and that steps are taken to mitigate bias. This may involve developing fairness-aware algorithms or, as a crucial future direction, incorporating social determinants of health and environmental factors as explicit model inputs to create more context-aware and equitable predictive tools.^20^,^21^

### Maintenance and Monitoring: Ensuring Long-term Model Viability

A deployed multimodal risk model is not a static entity; it will likely require regular recalibration and updating as clinical practice patterns, population characteristics, and treatment efficacies change over time. For example, as preventive therapies improve, baseline population risk may decrease, necessitating model adjustments to avoid overpredicting risk. Monitoring a model’s performance post-deployment and having a clear methodological framework for retraining or adjusting it are key components of safe and effective use. This requires an ongoing data collection, curation, and model governance infrastructure.

Taken together, while multimodal fusion models show great promise, they also embody the principle that “with great power comes greater responsibility.”^1^ The biomedical informatics field must navigate these technical hurdles of data integration, ensure robust methodological validation to move beyond hype from underpowered studies, and address the practical and ethical issues of implementation. Many of these challenges mirror those seen in any AI application in healthcare but are amplified by the complexity of dealing with multiple, heterogeneous data types. Recognizing these limitations provides a clear roadmap for future research, improvement, and the careful translation of these advanced models from research settings to actual clinical benefit.

## FUTURE PERSPECTIVES AND DIRECTIONS: ADVANCING THE INFORMATICS FRONTIER

The coming years are likely to witness significant advancements in multimodal data fusion for CAD risk prediction, moving from retrospective validation to impactful clinical tools. This progression will be driven by methodological innovations and informatics breakthroughs, demanding novel approaches from AI researchers and biomedical informaticians. Some key future directions and opportunities include:

### Prospective Clinical Trials and Implementation Studies: Methodological Imperatives for Real-world Validation

To truly assess the impact of multimodal risk models, rigorous testing in prospective clinical settings is essential. Methodologically, such trials must extend beyond predictive accuracy metrics to evaluate improvements in patient outcomes (e.g. fewer heart attacks) and cost-effectiveness when these AI-driven models guide interventions. For informaticians and trial designers, a key challenge lies in developing robust frameworks for seamlessly integrating these complex models into diverse clinical workflows and evaluating their real-world utility and adoption barriers through rigorous implementation science, an important allied field of informatics.

### Broader Data Integration: Expanding the Informatics Scope to “Total Lifestyle” and Environment

Future models will likely seek to incorporate data beyond the traditional medical sphere, presenting new informatics challenges and opportunities in data representation, linkage, and modeling. As noted in a recent editorial, linking social determinants of health and environmental factors (e.g. neighborhood deprivation, air pollution) can enrich risk predictions.^1^ Methodologically, this requires developing novel informatics techniques to quantitatively capture, harmonize, and integrate these highly heterogeneous, often unstructured or sparsely available, non-medical data streams with existing clinical and molecular data. For AI developers, creating models that can effectively learn from and reason over such diverse and causally complex data (e.g. by incorporating geospatial analysis or social determinants of health ontologies^20^,^21^) represents a significant research frontier toward a truly holistic, 360° patient view.

### Real-time Risk Monitoring and “Digital Twins”: Methodological Advancements in Dynamic Prediction

With increasing streaming data from wearables and continuous EHR updates, the concept of a dynamic, continuously learning risk score is becoming methodologically feasible. The cardiovascular “digital twin”—a virtual, dynamic model of an individual patient—could simulate intervention effects for personalized planning.^5^,^30^,^52^ From an informatics perspective, realizing this vision necessitates significant methodological breakthroughs in: (1) robust real-time streaming data analytics for noisy, high-velocity wearable-device data; (2) continual learning algorithms that allow models to adapt to evolving patient states without catastrophic forgetting; and (3) hybrid modeling approaches that can effectively integrate mechanistic physiological models with data-driven AI to ensure both predictive accuracy and clinical plausibility. This presents a rich area for AI research.

### Advanced ML Techniques: The Next Wave of Methodological Innovation

Methodologically, the field will see increased adoption and refinement of advanced ML techniques, demanding innovation from AI researchers. Transfer learning needs to evolve beyond simple fine-tuning to enable more effective knowledge adaptation across diverse cardiovascular datasets and tasks, especially in low-data regimes. Multitask learning frameworks could be designed to simultaneously predict a spectrum of related cardiovascular outcomes, potentially uncovering shared underlying pathways and improving model efficiency. Continual learning must address the stability–plasticity dilemma more effectively for dynamic risk models. Advanced generative models (e.g. diffusion models, advanced generative adversarial networks) offer promise for sophisticated data augmentation and realistic imputation of missing modalities but require methodological safeguards against generating misleading or biased synthetic data. A critical unmet need is the deeper integration of causal inference techniques with AI/ML; current models excel at correlation, but moving toward identifying modifiable, causal risk factors requires novel methods that combine observational data with causal discovery algorithms or allow for “what-if” scenario modeling beyond simple prediction. Furthermore, federated learning architectures need to become more robust, secure, and communication-efficient to enable collaborative model training on large, distributed datasets while rigorously preserving privacy and handling statistical heterogeneity across sites.

### Personalized Prevention through Precise Risk Stratification

As multimodal prediction methodologies more accurately identify high-risk individuals, they enable more aggressive or precisely tailored preventive strategies. Methodologically, the challenge shifts from mere prediction to prescription: developing AI systems that can not only forecast risk but also recommend optimal, individualized intervention strategies based on a patient’s unique multimodal profile and predicted response. This involves creating models that can learn from interventional data or employ reinforcement learning techniques to suggest therapies most likely to yield benefit for specific patient sub-phenotypes, thus truly operationalizing precision prevention.

### Clinical Implementation and Workflow Integration

**Figure 3:**
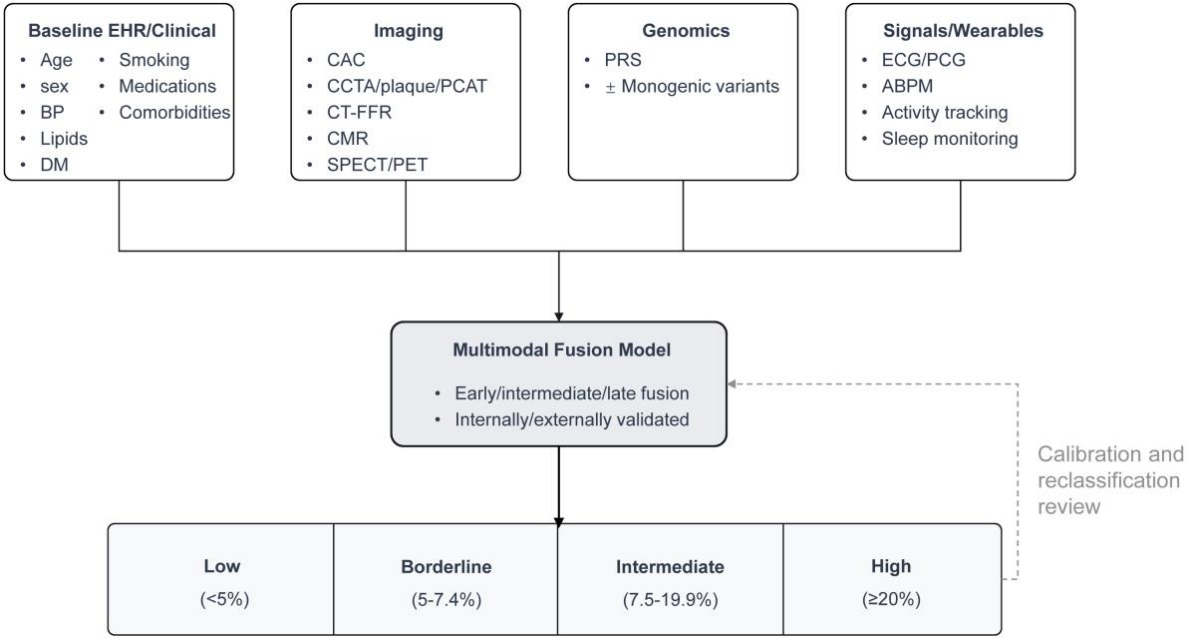
Clinical Workflow for Multimodal CAD Risk Stratification. Data from four key domains (Baseline EHR/Clinical, Imaging, Genomics, and Signals/Wearables) are integrated into a validated multimodal fusion model. The model outputs actionable risk strata, guiding personalized clinical decisions as outlined in the practical notes. Practical Notes: • CAC=0 may support deferring statins in low/intermediate risk • Very high PRS may prompt earlier imaging/intensification • Wearable-device alerts trigger clinical review but not autonomous therapy • Ensure fairness and consider federated learning for privacy ABPM, ambulatory blood pressure monitoring; BP, blood pressure; CAC, coronary artery calcium; CCTA, coronary computed tomography angiography; CMR, cardiac magnetic resonance; CT-FFR, computed tomography-derived fractional flow reserve; DM, diabetes mellitus; ECG, electrocardiogram; EHR, electronic health record; PCAT, pericoronary adipose tissue; PCG, phonocardiogram; PET, positron emission tomography; PRS, polygenic risk score; SPECT, single-photon emission computed tomography.

For multimodal models to transition from research concepts to clinical tools, their integration into established clinical workflows is paramount. This represents a significant informatics, human–computer interaction, and trust-building challenge that extends beyond mere technical embedding into the EHR. A potential workflow is conceptualized in Figure 3, which outlines how diverse data streams can be synthesized into actionable risk strata to guide clinical decision-making.

Methodologically, the challenge shifts from mere prediction to prescription. The true clinical utility of these models lies in their ability to inform personalized preventive strategies. For instance, as outlined in Figure 3, a coronary artery calcium (CAC) score of zero in a low- or intermediate-risk individual could support a shared decision-making conversation to defer or delay statin therapy.^28^ Conversely, a very high PRS, representing a significant lifelong genetic burden, could justify earlier and more aggressive primary prevention, such as initiating lipid-lowering therapy at a younger age or prompting referral for screening CCTA, even before traditional risk factors manifest.^41^,^44^

Longitudinal EHR-based ML models, such as those by Li et al.,^12^ offer a pathway to more dynamic risk assessment, potentially flagging patients whose risk trajectory is accelerating based on repeated measurements. Furthermore, alerts from wearable devices, while not yet ready for autonomous therapeutic action, could trigger timely clinical review for patients exhibiting concerning physiological trends.

However, significant implementation barriers remain, including workflow disruption, physician alert fatigue, and the practicalities of cost and accessibility. Advanced modalities like CCTA, CMR, and genomic testing are not universally available, particularly in lower-resource settings. This creates a risk that the benefits of multimodal AI may be limited to well-resourced academic centers, potentially exacerbating the health disparities discussed previously. Therefore, future research must focus not only on model accuracy but also on developing intuitive clinical decision support interfaces, robust explainability methods (explainable AI) tailored to clinician needs, and cost-effectiveness analyses to ensure these powerful tools can be equitably and effectively deployed at scale.

### Continuous Evaluation and Model Governance: Ensuring Trustworthy and Adaptive AI

Deployed multimodal AI models require robust systems for ongoing evaluation, governance, and adaptation. This includes periodic audits for performance drift, fairness, and potential biases across diverse populations. Methodological frameworks are needed for: (1) dynamic model updating or retraining as clinical practices, population characteristics, or even data sources evolve, without requiring complete redevelopment; (2) rigorous post-deployment surveillance to detect unexpected model behavior or errors; and (3) establishing clear “human-in-the-loop” protocols that define clinician oversight, responsibility, and model overriding capabilities. This “ModelOps” aspect of AI in healthcare is a critical informatics research area.

To summarize, the future of CAD risk prediction is trending toward holistic, individualized risk profiling, driven by informatics innovation. Multimodal data fusion is at the heart of this transformation. Achieving the aspiration of precise, preventative, and personalized CAD care within the next 5–10 years will require intensive, collaborative research between data scientists, AI methodologists, clinicians, and health systems, focusing on overcoming the outlined methodological and informatics challenges.

## CONCLUSION

Multimodal data fusion represents a significant methodological paradigm shift in CAD risk prediction, propelling the field from coarse, population-level estimates toward precise, individualized forecasts of patient risk, driven fundamentally by innovations in AI and biomedical informatics. This review has critically synthesized the informatics approaches and evaluated the evolving methodological landscape that underpins this transition over the past 15 years. By systematically deconstructing how diverse data modalities—imaging, genomics, EHR, and wearables—are being integrated through increasingly sophisticated AI and ML techniques, we have highlighted the complementary strengths harnessed and the demonstrable, albeit sometimes modest, improvements in predictive performance achieved over single-modality models.

More importantly from an informatics perspective, this review underscores that the true value of multimodal fusion lies not just in incremental AUC gains, but in its methodological capacity to model the multifactorial nature of CAD, capture complex non-linear interactions, and integrate longitudinal data dynamics—capabilities often beyond traditional risk assessment tools. We have identified key methodological patterns, from the utility of specific AI architectures like deep and graph neural networks for automated feature learning and cross-modal interaction modeling, to the distinct roles of various data types, such as PRS providing baseline genetic predisposition and EHRs offering rich temporal context.

However, the realization of this paradigm’s full potential is contingent upon the biomedical informatics community addressing substantial and ongoing methodological challenges. These include developing robust solutions for data heterogeneity, missingness, and algorithmic bias; enhancing model interpretability to foster clinical trust and utility; and establishing rigorous frameworks for prospective validation and seamless clinical workflow integration. These hurdles represent critical areas for future AI research and methodological innovation.

In summary, multimodal data fusion for CAD risk prediction serves as a compelling exemplar of AI’s transformative power in medicine and is a vibrant, rapidly advancing subfield of biomedical informatics. It directly aligns with the goals of precision medicine: delivering the right intervention to the right patient at the right time. By continuing to refine informatics methodologies for robustly maximally utilizing the totality of patient data, the AI in medicine community can significantly enhance risk prediction, personalize preventive strategies, and ultimately reduce the global burden of CAD. While the journey from complex data to actionable clinical wisdom is ongoing and demanding, the trajectory toward more informed, precise, and AI-driven individualized care for patients at risk of CAD is firmly established.

## Supplementary Information



## Abbreviations

AI: artificial intelligence
AUC: area under the curve
CAC: coronary artery calcium
CAD: coronary artery disease
CCTA: coronary computed tomography angiography
CI: confidence interval
C-index: concordance index
CMR: cardiac magnetic resonance
CNNs: convolutional neural networks
CT-FFR: computed tomography-derived fractional flow reserve
ECG: electrocardiogram
EHRs: electronic health records
GCN: graph convolutional network
IDI: integrated discrimination improvement
MESA: multi-ethnic study of atherosclerosis
ML: machine learning
PROBAST: prediction model risk of bias assessment tool
PRS: polygenic risk score
SPECT: single-photon emission computed tomography.
