# Supplementary material for: A Narrative Review of Multimodal Data Fusion Strategies for Precision Risk Prediction in Coronary Artery Disease: Advances, Challenges, and Future Informatics Directions
Source: Rambam Maimonides Med J. 2025 Oct 31;16(4):e0023. doi: 10.5041/RMMJ.10558 (PMC12591514; doi:10.5041/RMMJ.10558)
Supplement: Supplementary file 1 [file rmmj-16-4-e0023_Supplement.pdf]

SUPPLEMENTARY MATERIAL

This appendix has been provided by the authors for the benefit of readers

Supplement to A Narrative Review of Multimodal Data Fusion Strategies for Precision Risk Prediction in Coronary Artery Disease: Advances, Challenges, and Future Informatics Directions

Zhou Z, Wang J. A Narrative Review of Multimodal Data Fusion Strategies for Precision Risk Prediction in Coronary Artery Disease: Advances, Challenges, and Future Informatics Directions. Rambam Maimonides Med J 2025;16 (4):e0023. Review. doi:10.5041/RMMJ.10558

SUPPLEMENTARY TABLE 1: SUMMARY OF 39 MULTIMODAL FUSION STUDIES FOR CAD RISK PREDICTION

This supplement summarizes 39 empirical studies on multimodal data fusion for coronary artery disease (CAD) risk prediction, published between 2009 and 2025. The table highlights the diversity of integrated data modalities and analytical approaches. Particularly noteworthy is the consistent, albeit modest, improvement in predictive performance (discrimination and reclassification) when imaging or genomic data are fused with traditional clinical risk factors. Furthermore, the included studies showcase a methodological trend towards adopting advanced machine learning algorithms and validating models on external cohorts, signaling a maturation of the field. This comprehensive summary serves as a detailed evidence base for the main manuscript.

The references to this table are provided after the table.

Table S1. Summary of 39 Multimodal Fusion Studies for CAD Risk Prediction.

First author (year)	Country/ setting	*N* (events)	Modalities (details)	Endpoint and horizon	Fusion strategy	Algorithm(s)	Validation	AUC/C-index (95% CI)	Calibration	Reclassification (NRI/IDI)	PROBAST overall (L/M/H)**
Motwani et al. (2017)[1]	International/ CONFIRM registry (17 centers)	10,030 pts (745 deaths over 5 years)	CCTA (44 features) + clinical (25 features)	All-cause mortality/5 years	Early (feature-level fusion)	Boosted ensemble ML (information gain + LogitBoost)	10-fold stratified cross-validation	AUC = 0.79 (vs FRS = 0.61, SIS = 0.64, DI = 0.62)	NR	NR; ML outperformed all comparators	M: strong design, lacks external validation
Betancur et al. (2018)[2]	US/Cedars-Sinai + multicenter registry	2,619 pts (239 MACE over 3.2 ± 0.6 years)	SPECT MPI (25 imaging vars) + stress test (17 vars) + clinical (28 vars)	MACE (death, MI, UA, late revasc)/3 years	Early (feature-level fusion)	Boosted ensemble ML (info gain + LogitBoost)	10-fold stratified cross-validation	AUC 0.81 vs imaging 0.78	Calibration curves reported	NRI = 26% vs MD diagnosis (*P* < 0.001)	M: strong design, lacks external validation
Coenen et al. (2018)[3]	5-center (EU/US/Asia)	351 pts/ 525 vessels (FFR reference)	CCTA + ML-derived CT-FFR	Functionally significant CAD (diagnostic)	Late (ML applied to CCTA-derived features)	Deep learning model for CT-FFR	Internal (per-vessel and per-patient analysis)	AUC = 0.84 (ML-CT-FFR); CTA alone = 0.69	NR	73% of CTA false positives correctly reclassified	M: strong design, lacks external validation
Inouye et al. (2018)[4]	UK/UK Biobank	22,242 CAD cases/ 460,387 non-cases	Genomic (metaGRS from 1.7M variants) + Clinical (traditional risk factors)	Incident CAD/median 8 years	Late (model-level) fusion: GRS added to clinical risk factor model	Cox proportional hazards	External (metaGRS in UK Biobank)	C-index (Clinical-only): 0.641 (0.633-0.649) C-index (Clinical + GRS): 0.655 (0.647-0.663)	Adequate; stratified cumulative risk curves reported	Reported; significantly reclassified individuals into different risk trajectories	M: strong cohort, landmark GRS validation, fusion is an additive (late) model
Tesche et al. (2018)[5]	US/Medical University of South Carolina	85 pts/ 159 lesions (FFR ≤0.80)	CCTA-derived FFR via ML vs CFD	Lesion-specific ischemia/ diagnostic	Single-modality algorithmic comparison	ML-based FFR vs CFD-based FFR	Internal (retrospective cohort)	AUC ~0.84 vs 0.69 CTA	FFR-ML: AUC = 0.89; FFR-CFD: AUC = 0.89; CCTA: AUC = 0.61	NR	M: strong design, lacks multimodal integration
Zhao et al. (2019)[6]	US/Vanderbilt University Medical Center	109,490 adults (CVD events NR)	Longitudinal EHR + genetic data	First CVD event/10 years	Late (genetic + EHR via model-level fusion)	LR, RF, GBT, CNN, LSTM	Nested 10-fold CV	Best AUROC = 0.79 (LSTM); baseline ACC/AHA = 0.73	NR	NR, DL outperformed baseline	M: strong design, lacks external validation
Alaa et al. (2019)[7]	UK/UK Biobank	423,604 (4,801 CVD events)	Structured clinical + lifestyle + self-reported variables (473 total)	First CVD event/5 years	Early (AutoML feature-level fusion)	AutoPrognosis (ensemble ML pipeline)	Internal only	AUC = 0.774 (95% CI: 0.768-0.780)	Included in pipeline	NR; 368 more cases predicted vs Framingham	M

First author (year)	Country/ setting	*N* (events)	Modalities (details)	Endpoint and horizon	Fusion strategy	Algorithm(s)	Validation	AUC/C-index (95% CI)	Calibration	Reclassification (NRI/IDI)	PROBAST overall (L/M/H)**
Al'Aref et al. (2020)[8]	Multinational/ CONFIRM registry	13,054 pts (2,380 obstructive CAD)	Clinical (25 vars) + CACS	Obstructive CAD on CCTA/cross-sectional	Early (feature-level fusion)	XGBoost (boosted ensemble ML)	80/20 split + 10-fold CV	ML+CACS AUC = 0.881; ML alone = 0.773; CAD consortium = 0.734; UDF = 0.682	Calibration slopes reported	Net reclassification improvement shown graphically	M: strong design, lacks external validation
Zhang et al. (2020)[9]	China/single-center hospital	62 (32 CAD, 30 CPNCA)	ECG (multi-domain) + PCG (multi-domain) + Holter + echocardiography + biomarkers	CAD detection/ cross-sectional	Early (feature-level)	Hybrid feature selection (MI, RFE, RF, SVM weights) + SVM	Nested CV	Accuracy = 96.67%; SN = 96.67%; SP = 96.67%; F1 = 96.64%	NR	NR	M: small sample size, no external validation, but methodologically complete
Elliott (2020)[10]	UK/UK Biobank	352,660 (6,272 incident CAD events over 8 years)	Clinical risk score (PCE) + PRS	Incident CAD/median 8 years	Late (model-level fusion: PCE + PRS)	Logistic regression + PRS integration	I Independent cohort (UK Biobank)	PCE = 0.76, PRS = 0.61, Combined = 0.78	Recalibrated; PCE alone over-estimated risk	Net reclassification improvement = 4.0% (95% CI: 3.1%-4.9%)	M: strong design, modest gain
Pickhardt et al. (2020)[11]	US/NIH + University of Wisconsin	9,223 asymptomatic adults (1,831 CV events or deaths over median 8.8 years)	Abdominal CT-derived biomarkers: aortic calcification, muscle density, fat ratio, liver fat, vertebral BMD	Major CV events + all-cause mortality/2-10-year follow-up	Early (feature-level fusion of CT biomarkers)	Deep learning + feature-based extraction	Internal cohort; no external validation	Combined AUROC = 0.811 (2-year survival); individual CT biomarkers outperform FRS/BMI	Adequate; hazard ratios by quartile reported	Not formally reported; CT biomarkers significantly better than FRS/BMI	M: strong imaging design, lacks multimodal integration
Kwan et al. (2021)[12]	US/Cedars-Sinai + dual-center registry	352 pts/ 1056 vessels (26% revascularized)	CCTA quantitative plaque features + clinical risk factors	Revasc (PCI or CABG)/ within 3 months	Late (ML-IRS added to clinical + stenosis model)	ML-IRS	Internal cohort; no external validation	Traditional model: 0.69 → ML-enhanced: 0.78 (*P* < 0.0001)	Adequate; ML-IRS stratified by revasc status	NRI = 0.636 (95% CI: 0.503-0.769)	M: strong imaging design, lacks external validation
Sun et al. (2021)[13]	UK/UK Biobank	306,654 individuals /NR CVD events over 8.1 years	PRS + conventional clinical risk factors	First-onset CVD/median 8.1 years	Late (model-level fusion: PRS + clinical)	Cox proportional hazards	Internal cohort; population-level modeling	C-index: 0.710 → 0.722 (+0.012)	Adequate; cumulative incidence curves reported	NRI: ~10% (cases), ~12% (non-cases)	M: strong cohort, limited modeling innovation

First author (year)	Country/ setting	*N* (events)	Modalities (details)	Endpoint and horizon	Fusion strategy	Algorithm(s)	Validation	AUC/C-index (95% CI)	Calibration	Reclassification (NRI/IDI)	PROBAST overall (L/M/H)**
Li et al. (2021)[14]	China/ PhysioNet multi-center dataset	388 recordings (expanded to ~1975 segments; balanced)	ECG (8s, 2kHz) + PCG (8s, 1kHz, 4 frequency bands)	CHD, T2D, metabolic health status/cross-sectional	Early (feature-level)	CL-ECG-Net, CL-PCG-Net, GA, SVM	5-fold CV × 10 repeats	0.936 (multi-modal GA+SVM)	NR	NR	M: strong design, lacks external validation, limited dataset
Barbieri et al. (2022)[15]	New Zealand/ Nationwide administrative data	2,164,872 (61,927 CVD events)	Linked administrative data: diagnoses, medications, encounters	First CVD event/5 years	Early (structured feature-level fusion)	DeepSurv (DL survival) vs Cox	Internal (sex-specific models)	R^2: 0.468 (DL) vs 0.425 (Cox) in women	Good (*P* < 0.0001)	NR	M: lacks external validation
Miller et al. (2022)[16,17]	US (Cedars-Sinai + multicenter reader study)	240 pts (50% obstructive CAD by ICA)	SPECT MPI + physician interpretation + DL model output	Obstructive CAD (≥50% LM or ≥70% other segments)/ diagnostic	Late (reader + DL fusion)	CAD-DL (explainable DL model trained on separate cohort)	Reader study (3 physicians with/without DL support)	Physician alone: 0.747; Physician + DL: 0.779; DL alone: 0.78	NR	NRI = 17.5% (95% CI: 9.8%-24.7%) with DL support	M: strong design, lacks external validation
Lin et al. (2022)[18]	US + Netherlands/ PACIFIC trial	208 pts/581 vessels (FFR ≤0.80: 139 vessels; MBF ≤2.30: 195 vessels)	CCTA quantitative plaque features + PET MBF + invasive FFR	Vessel-specific ischemia (FFR) + impaired MBF/ diagnostic	Early (feature-level fusion of CCTA metrics)	ML model trained on NXT trial data	External validation (PACIFIC trial)	FFR prediction: AUC = 0.92; MBF prediction: AUC = 0.80	Calibration plots reported; good agreement	NR; ML outperformed visual reads	M: strong external validation, limited clinical variables
King et al. (2022)[19]	UK/UK Biobank	272,307 individuals /7,036 incident CAD cases	Integrated PRS + pooled cohort clinical variables	Incident CAD/12-year follow-up	Late (model-level fusion: PRS-enhanced PCE)	Cox proportional hazards	Internal cohort; no external validation	PRS-enhanced PCE: 0.753 (95% CI: 0.748-0.758); PCE alone: 0.718	Adequate; cumulative risk curves reported	NRI = 9.3% overall (cases: +11.7%; non-cases: -2.3%)	M: strong cohort, lacks external validation
Vassy et al. (2023)[20]	US/Million Veteran Program	79,151 individuals /5,485 ASCVD events	Genome-wide PRS (CAD + stroke) + traditional clinical risk factors	Incident ASCVD (MI, stroke, death)/ median 4.3 years	Late (model-level fusion: PRS + clinical)	Cox proportional hazards	Internal cohort; multi-ancestry stratified analysis	NR; modest improvement in discrimination	Adequate; cumulative incidence curves reported	NRI modest: 0.38% (men), 6.79% (women), age-stratified	M: strong cohort, limited modeling innovation
Khan et al. (2023)[21]	US + Netherlands/ MESA + Rotterdam Study	3,208 participants (incident CHD over 10 years)	Clinical risk score (PCE) + CACS + PRS	Incident CHD/10-year follow-up	Late (model-level fusion: PCE + CACS + PRS)	Cox regression + additive risk modeling	Independent cohorts (MESA, RS)	CACS alone: 0.76; PRS alone: 0.69; Combined: 0.78	Adequate (x^2 < 20 for all models)	NRI: +0.19 for CACS, +0.04 for PRS (only CACS significant)	M: strong design, PRS contribution modest

First author (year)	Country/ setting	*N* (events)	Modalities (details)	Endpoint and horizon	Fusion strategy	Algorithm(s)	Validation	AUC/C-index (95% CI)	Calibration	Reclassification (NRI/IDI)	PROBAST overall (L/M/H)**
Zambrano et al. (2023)[22]	US/Stanford Health System	8,139 pts (IHD events over 5 years; exact count NR)	Abdominopelvic CT (body composition features) + EMR (labs, vitals, diagnoses)	Incident ischemic heart disease/5-year follow-up	Early (feature-level fusion of CT + EMR)	Gradient boosting + SHAP explainability	Internal split (train/test); no external cohort	Combined model AUROC = 0.81; EMR-only = 0.78; CT-only = 0.76	Calibration curves reported; good agreement	NR, model outperformed PCE and Framingham scores	M: strong design, lacks external validation
Pujadas et al. (2023)[23]	UK/UK Biobank	NR; incident AF, HF, MI, stroke over longitud-inal follow-up	Clinical risk factors (VRF) + CMR indices + CMR radiomics	Incident CVDs (AF, HF, MI, stroke)/ multi-year follow-up	Early (feature-level fusion of VRF + CMR + radiomics)	SVM	Internal split; no external cohort	HF: AUC = 0.84 (VRF+CMR+ Rad); AF: AUC = 0.76; MI/stroke: lower	NR	NR; radiomics showed incremental value	M: strong design, lacks external validation
Durmaz et al. (2023)[24]	Turkey/Single-center)	60 STEMI pts	LGE + cine CMR radiomics + clinical + CMR params	MACE/follow-up period NR	Early (feature-level)	ML (NN, RF, SVM, NB, etc.)	Split-sample + repeated random sampling	Best model AUC = 0.965	NR	Added value vs clinical/CMR models (qualitative)	L: small sample, no external validation
Li et al. (2024)[25]	China/CHERRY cohort	215,744 (6,081 ASCVD)	Demographics (age, sex, education), medication use, repeated labs (lipids, glycemia), BP, obesity indices, renal function	5-year ASCVD (non-fatal MI, CHD death, fatal/non-fatal stroke), 6,081 events	Early (feature-level)	XGBoost (tree-based boosting), LASSO regression; compared with refitted China-PAR Cox model	Internal split (train/test), large-scale EHR cohort (*n*=215,744)	C-stat = 0.792 (XGB), 0.789 (LASSO)	Hosmer-Lemeshow *P*>0.05; calibration curves	NRI 3.9% (1.4%-6.4%), 2.8% (0.7%-4.9%)	M
Bock et al. (2024)[26]	Switzerland (BASEL VIII study)	3,522 (fCAD prevalence = 32.9%)	Stress ECG signals + 8 clinical vars + cardiologist VAS	fCAD diagnosis/ cross-sectional	Late (logistic fusion of DL + ML + clinician VAS)	CARPE ECG (ResNet + multitask) + CARPE Clin. (RF) + CARPE Coll. (logistic fusion)	Internal temporal split (75/25) + external validation (THEW cohort)	Internal: ECG = 0.71, Clinical = 0.70, Combined = 0.74	Bootstrapped CIs; DCA curves reported	Imaging reduction: up to 17.3% (CARPE Coll. vs cardiologist)	M: strong design, no prospective impact analysis
Bock et al. subgroup (2024)[26]*	Israel (external validation cohort)	916 (fCAD prevalence = 7.5%)	Treadmill ECG + same 8 clinical vars	fCAD diagnosis/ cross-sectional	Late (same as main study)	CARPE ECG + CARPE Clin.	External (THEW cohort)	ECG = 0.80 ± 0.01, Clinical = 0.75 ± 0.004	Good; bootstrapped CIs	NR	M: external only

First author (year)	Country/ setting	*N* (events)	Modalities (details)	Endpoint and horizon	Fusion strategy	Algorithm(s)	Validation	AUC/C-index (95% CI)	Calibration	Reclassification (NRI/IDI)	PROBAST overall (L/M/H)**
Chen et al. (2024)[27]	China/Suzhou Medical Association	608 CAD pts/ NR MACE count	Lesion-specific PCAT radiomics (CT-FFR guided) + clinical variables	MACE (CV death, MI, revasc, UA hospital-ization)/ retrospective	Late (model-level fusion: clinical + Rad-score + CT-FFR)	LASSO + multivariable Cox regression	Internal cohort; no external validation	Combined model: C-index = 0.718; AUC = 0.773	Adequate; Kaplan-Meier and calibra-tion curves reported	NR; incremental gain shown	M: strong imaging design, lacks external validation
Yang et al. (2024)[28]	China/ Shanghai General Hospital	1,392 diabetic pts (108 MACE over 5 years)	Clinical + CCTA high-risk plaque features + PCAT radiomics	MACE/5-year follow-up	Late (model-level fusion: clinical + imaging + radiomics)	Multivariable Cox regression	Internal split (training: 835; validation: 557)	Model-1: 0.68; Model-2: 0.79; Model-3: 0.80 (*P* = 0.408 vs Model-2)	Adequate; calibration curves reported	NR; PCAT radiomics not incrementally predictive	M: strong design, lacks external validation
Romero-Farina et al. (2024)[29]	Spain/multi-center cohort	2,226 women (148 MACE in training set)	Clinical variables + stress test data + gSPECT MPI parameters	4-year MACE prediction	Early (feature-level)	Cox regression (CORSWO risk score)	Internal split (train/ validation)	Training AUC = 0.80 (95% CI: 0.74-0.83); Validation AUC = 0.78 (95% CI: 0.70-0.83)	Brier score = 0.08; calibration curves reported	NR	M: internal validation only; no external cohort; meth-odologically robust
Wang et al. (2024)[30]	China/Public datasets (5 combined)	NR (from 5 CAD datasets)	Tabular clinical data (demographics, symptoms, labs)	CAD diagnosis/ cross-sectional	Ensemble AutoML (AutoGluon)	AutoML ensemble + SHAP for explainability	4-fold cross-bagging	AUC = 0.9562, ACC = 0.9167	SHAP-based feature attribution; calibration curve	NR; ensemble outperformed baselines	M: strong performance, lacks external validation
Wang et al. (2024)[31]	China/Multi-center	294 (CABG pts, MACE endpoint)	Clinical (52 vars) + NT-proBNP + SII + echocardiogra-phy	MACE/long-term follow-up	Early (feature-level fusion)	LASSO + Cox regression	External validation cohort (*n*=118)	C-index: 0.768 (dev), 0.633 (val)	Good (calibration curves)	NR	M: lacks reclassification metrics
Zhan et al. (2024)[32]	China/North Sichuan Medical College	239 angina pts (46 MACE)	CCTA-derived PCAT radiomics + FAI + clinical variables	MACE (CV death, MI, revasc, UA hospitaliza-tion)/ retrospective	Late (model-level fusion: clinical + FAI + radiomics)	ML-based logistic regression (feature selection method NR)	Internal split (train: 167; validation: 72)	Radiomics model: AUC = 0.83 (train), 0.71 (validation); *P* < 0.05 vs others	Good; calibration curves reported	NR, radiomics model showed incremental gain	M: strong imaging design, lacks external validation
Badawy et al. (2025)[33]	Egypt/Public datasets (UCI repository)	NR (combined from 4 datasets)	Multi-source tabular clinical data	CAD diagnosis/ cross-sectional	Late fusion via ensemble learning	LR, RF, XGB, SVM, NB, DT, KNN → ensemble model	Internal cross-validation	Accuracy = 98.46%, Recall = 100%, F1 = 98%	NR	NR; ensemble outperformed all single models	M: strong performance, lacks external validation

First author (year)	Country/ setting	*N* (events)	Modalities (details)	Endpoint and horizon	Fusion strategy	Algorithm(s)	Validation	AUC/C-index (95% CI)	Calibration	Reclassification (NRI/IDI)	PROBAST overall (L/M/H)**
Gabriel et al. (2025)[34]	US/Emory + Georgia Tech	25,514 pts (2.93% experienced MACE over 10 years)	CAC + ECG + lab + clinical risk scores	10-year MACE /retrospective	Late (sequential model-level integration)	XGBoost + SHAP	Internal cross-validation (5-fold × 10 seeds)	AUC = 0.883 ± 0.012	Good; decision curves and SHAP interpretation	NR; 30% gain over CAC	M: large cohort, strong modeling, preprint
Li et al. (2025)[35]	China/Multi-center (2 centers)	1,024 STEMI pts (169 MACE events)	Cine MRI radiomics + LVEF + LGE + clinical	MACE/median follow-up 3.1 years	Early (Rad-score + imaging + clinical)	Logistic regression + Cox model	External validation (205 pts)	AUC = 0.83 (train), 0.71 (test)	Good fit (calibration curves, $P > 0.05$)	Risk reclassification: 33% (train), 34% (test)	M: good design, lacks calibration stats
Li et al. (2025)[36]	China/ Prospective, dual-center	190 (CAD confirmed via CAG)	Oral microbiome (16sRNA) + tongue hyperspectral imaging	CAD diagnosis/ cross-sectional	Late fusion (GP-GB-SVM ensemble)	30 ML models + fusion model (Gaussian process + gradient boosting + SVM)	Internal + external test sets	AUC = 0.92 (internal), 0.86 (external)	Calibration curves reported (good fit)	Not reported; fusion model outperformed all single models	M: strong design, lacks long-term outcome
Pezel et al. (2025)[37]	France/ multicenter (Jacques Cartier, Lariboisière, American Hospital of Paris)	2,038 pts (281 MACE over 7 years)	CCTA plaque metrics + stress cardiac MRI + clinical + ECG	MACE (CV death + non-fatal MI)/median 7-year follow-up	Early (feature-level fusion of imaging + clinical + ECG)	LASSO + XGBoost (multimodal feature-level fusion)	Internal + 2 external cohorts	ML model: 0.86; external: 0.84 and 0.92	Good; calibration curves reported	NR; ML outperformed all comparators	M: strong design, excellent external validation
Zhang et al. (2025)[38]	China/ Shanghai University of Traditional Chinese Medicine	488 CAD pts (stenosis severity by ICA)	Facial morphometrics + tongue/lip images + pulse/ pressure waveforms + lab biomarkers	Coronary stenosis severity/ diagnostic	Early + adaptive weighting (transformer-based fusion)	Transformer + residual modules + adaptive fusion	Internal + external validation	Accuracy: 90% (train), 85% (external validation); AUC NR	NR	NR	M: innovative design, external validation present
Zou et al. (2025)[39]	China/multi-center	237 hypertensive CAD pts/ NR MACE	PCAT radiomics + CT-FFR + clinical features	MACE/2-year follow-up	Early (feature-level fusion)	LASSO + LDA	Internal split (train/test = 165/72)	AUC = 0.886 (train), 0.786 (test)	Good; calibration + decision curves	NR; specificity improved	M: strong imaging fusion, lacks external validation

See legend on following page.

* External validation from Bock et al. (2024).

** Risk of bias was assessed using the PROBAST tool across four domains—participants, predictors, outcomes, and analysis. Studies were rated as Low (L), Medium (M), or High (H) risk of bias. Most studies were rated Medium due to limitations such as lack of external validation, incomplete calibration reporting, or unclear fusion strategy definitions. Studies with high bias risk were excluded during screening.

Abbreviations: ACC/AHA, American College of Cardiology/American Heart Association; AF, atrial fibrillation; AI, artificial intelligence; ASCVD, atherosclerotic cardiovascular disease; AUC, area under the curve; AUROC, area under the receiver operating characteristic curve; BMD, bone mineral density; BMI, body mass index; BP, blood pressure; CABG, coronary artery bypass grafting; CACS, coronary artery calcium score; CAD, coronary artery disease; CAG, coronary angiography; CARPE, Coronary Artery disease Risk Prediction using ECG (study/model name); CCTA, coronary computed tomography angiography; CFD, computational fluid dynamics; CHD, coronary heart disease; CL-ECG-Net, Contrastive Learning Electrocardiogram Network; CL-PCG-Net, Contrastive Learning Phonocardiogram Network; China-PAR, Prediction for ASCVD Risk in China equation; CI, confidence interval; C-index, concordance index; CMR, cardiac magnetic resonance; CNN, convolutional neural network; CORSWO, Coronary Risk Score in Women; CT, computed tomography; CTA, computed tomography angiography; CV, cross-validation; CVD, cardiovascular disease; DCA, decision curve analysis; DI, Duke Index; DL, deep learning; DT, decision tree; ECG, electrocardiogram; EHR, electronic health record; EMR, electronic medical record; fCAD, functionally relevant CAD; FAI, fat attenuation index; FFR, fractional flow reserve; F1, F1-score (harmonic mean of precision and recall); FRS/BMI, Framingham Risk Score / body mass index; GA, genetic algorithm; GBT, gradient boosting trees; GP, Gaussian process; GB, gradient boosting; GRS, genomic risk score; gSPECT, gated single-photon emission computed tomography; HF, heart failure; HR, hazard ratio; ICA, invasive coronary angiography; IDI, integrated discrimination improvement; KNN, k-nearest neighbors; L, low risk of bias; LASSO, least absolute shrinkage and selection operator; LDA, linear discriminant analysis; LGE, late gadolinium enhancement; LM, left main coronary artery; LR, logistic regression; LSTM, long short-term memory; LVEF, left ventricular ejection fraction; M, medium risk of bias; MACE, major adverse cardiovascular events; MBF, myocardial blood flow; MD, medical doctor; MESA, Multi-Ethnic Study of Atherosclerosis; MI, myocardial infarction; ML, machine learning; ML-IRS, ML-based ischemic risk score; MPI, myocardial perfusion imaging; NB, Naïve Bayes; NR, not reported; NRI, net reclassification improvement; NXT trial, Next Steps Toward CT-FFR trial; PCAT, pericoronary adipose tissue; PCE, pooled cohort equations; PCG, phonocardiogram; PCI, percutaneous coronary intervention; PET, positron emission tomography; PMID, PubMed identifier; PROBAST, Prediction model Risk Of Bias Assessment Tool; PRS, polygenic risk score; pts, patients; Rad-score, radiomics score; revasc, revascularlization; RF, random forest; RFE, recursive feature elimination; RS, Rotterdam Study; SPECT, single-photon emission computed tomography; SN, sensitivity; SP, specificity; SHAP, SHapley Additive exPlanations; SIS: Segment Involvement Score; STEMI, ST-elevation myocardial infarction; SVM, support vector machine; T2D, type 2 diabetes; THEW, Telemetric and Holter ECG Warehouse; UA, unstable angina; UDF, updated Diamond–Forrester model; VAS, visual analog scale; VRF, conventional vascular risk factors; XGB, eXtreme Gradient Boosting (short form of XGBoost); XGBoost, eXtreme Gradient Boosting.

REFERENCES

1. Motwani M, Dey D, Berman DS, et al. Machine learning for prediction of all-cause mortality in patients with suspected coronary artery disease: a 5-year multicentre prospective registry analysis. Eur Heart J 2017;38:500–7. CrossRef
2. Betancur J, Otaki Y, Motwani M, et al. Prognostic value of combined clinical and myocardial perfusion imaging data using machine learning. JACC Cardiovasc Imaging 2018;11:1000–9. CrossRef
3. Coenen A, Kim YH, Kruk M, et al. Diagnostic accuracy of a machine-learning approach to coronary computed tomographic angiography-based fractional flow reserve: result from the MACHINE Consortium. Circ Cardiovasc Imaging 2018;11:e007217. CrossRef
4. Inouye M, Abraham G, Nelson CP, et al. Genomic risk prediction of coronary artery disease in 480,000 adults: implications for primary prevention. J Am Coll Cardiol 2018;72:1883–93. CrossRef
5. Tesche C, De Cecco CN, Baumann S, et al. Coronary CT angiography-derived fractional flow reserve: machine learning algorithm versus computational fluid dynamics modeling. Radiology 2018;288:64–72. CrossRef
6. Zhao J, Feng Q, Wu P, et al. Learning from longitudinal data in electronic health record and genetic data to improve cardiovascular event prediction. Sci Rep 2019;9:717. CrossRef
7. Alaa AM, Bolton T, Di Angelantonio E, Rudd JHF, van der Schaar M. Cardiovascular disease risk prediction using automated machine learning: a prospective study of 423,604 UK Biobank participants. PLoS One 2019;14:e0213653. CrossRef
8. Al'Aref SJ, Maliakal G, Singh G, et al. Machine learning of clinical variables and coronary artery calcium scoring for the prediction of obstructive coronary artery disease on coronary computed tomography angiography: analysis from the CONFIRM registry. Eur Heart J 2020;41:359–67. CrossRef
9. Zhang H, Wang X, Liu C, et al. Detection of coronary artery disease using multi-modal feature fusion and hybrid feature selection. Physiol Meas 2020;41. CrossRef
10. Elliott J, Bodinier B, Bond TA, et al. Predictive accuracy of a polygenic risk score-enhanced prediction model vs a clinical risk score for coronary artery disease. JAMA 2020;323:636–45. CrossRef
11. Pickhardt PJ, Graffy PM, Zea R, et al. Automated CT biomarkers for opportunistic prediction of future cardiovascular events and mortality in an asymptomatic screening population: a retrospective cohort study. Lancet Digit Health 2020;2:e192–e200. CrossRef
12. Kwan AC, McElhinney PA, Tamarappoo BK, et al. Prediction of revascularization by coronary CT angiography using a machine learning ischemia risk score. Eur Radiol 2021;31:1227–35. CrossRef
13. Sun L, Pennells L, Kaptoge S, et al. Polygenic risk scores in cardiovascular risk prediction: a cohort study and modelling analyses. PLoS Med 2021;18:e1003498. CrossRef
14. Li P, Hu Y, Liu Z-P. Prediction of cardiovascular diseases by integrating multi-modal features with machine learning methods. Biomed Signal Process Control 2021;66:102474. CrossRef
15. Barbieri S, Mehta S, Wu B, et al. Predicting cardiovascular risk from national administrative databases using a combined survival analysis and deep learning approach. Int J Epidemiol 2022;51:931–44. CrossRef
16. Miller RJH, Kuronuma K, Singh A, et al. Explainable deep learning improves physician interpretation of myocardial perfusion imaging. J Nucl Med 2022;63:1768–74. CrossRef
17. Miller RJH, Hauser MT, Sharir T, et al. Machine learning to predict abnormal myocardial perfusion from pre-test features. J Nucl Cardiol 2022;29:2393–403. CrossRef
18. Lin A, van Diemen PA, Motwani M, et al. Machine learning from quantitative coronary computed tomography angiography predicts fractional flow reserve-defined ischemia and impaired myocardial blood flow. Circ Cardiovasc Imaging 2022;15:e014369. CrossRef
19. King A, Wu L, Deng HW, Shen H, Wu C. Polygenic risk score improves the accuracy of a clinical risk score for coronary artery disease. BMC Med 2022;20:385. CrossRef

20. Vassy JL, Posner DC, Ho YL, et al. Cardiovascular disease risk assessment using traditional risk factors and polygenic risk scores in the Million Veteran Program. JAMA Cardiol 2023;8:564–74. CrossRef

21. Khan SS, Post WS, Guo X, et al. Coronary artery calcium score and polygenic risk score for the prediction of coronary heart disease events. JAMA 2023;329:1768–77. CrossRef

22. Zambrano Chaves JM, Wentland AL, Desai AD, et al. Opportunistic assessment of ischemic heart disease risk using abdominopelvic computed tomography and medical record data: a multimodal explainable artificial intelligence approach. Sci Rep 2023;13:21034. CrossRef

23. Pujadas ER, Raisi-Estabragh Z, Szabo L, et al. Prediction of incident cardiovascular events using machine learning and CMR radiomics. Eur Radiol 2023;33:3488–500. CrossRef

24. Durmaz ES, Karabacak M, Ozkara BB, et al. Radiomics-based machine learning models in STEMI: a promising tool for the prediction of major adverse cardiac events. Eur Radiol 2023;33:4611–20. CrossRef

25. Li C, Liu X, Shen P, et al. Improving cardiovascular risk prediction through machine learning modelling of irregularly repeated electronic health records. Eur Heart J Digit Health 2024;5:30–40. CrossRef

26. Bock C, Walter JE, Rieck B, et al. Enhancing the diagnosis of functionally relevant coronary artery disease with machine learning. Nat Commun 2024;15:5034. CrossRef

27. Chen M, Hao G, Xu J, et al. Radiomics analysis of lesion-specific pericoronary adipose tissue to predict major adverse cardiovascular events in coronary artery disease. BMC Med Imaging 2024;24:150. CrossRef

28. Yang W, Ding X, Yu Y, et al. Long-term prognostic value of CT-based high-risk coronary lesion attributes and radiomic features of pericoronary adipose tissue in diabetic patients. Clin Radiol 2024;79:931–40. CrossRef

29. Romero-Farina G, Aguadé-Bruix S, Ferreira-González I. Prediction of major adverse coronary events using the coronary risk score in women. Radiol Cardiothorac Imaging 2024;6:e230381. CrossRef

30. Wang J, Xue Q, Zhang CWJ, Wong KKL, Liu Z. Explainable coronary artery disease prediction model based on AutoGluon from AutoML framework. Front Cardiovasc Med 2024;11:1360548. CrossRef

31. Wang J, Wang Y, Duan S, et al. Multimodal data-driven prognostic model for predicting long-term prognosis in patients with ischemic cardiomyopathy and heart failure with preserved ejection fraction after coronary artery bypass grafting: a multicenter cohort study. J Am Heart Assoc 2024;13:e036970. CrossRef

32. Zhan W, Luo Y, Luo H, et al. Predicting major adverse cardiovascular events in angina patients using radiomic features of pericoronary adipose tissue based on CCTA. Front Cardiovasc Med 2024;11:1462451. CrossRef

33. Badawy M, Ramadan N, Hefny HA. Toward reliable coronary heart disease prediction: integrating multi-source data with ensemble machine learning. J Imaging Inform Med 2025;August 15: online ahead of print. CrossRef

34. Gabriel RM, van Assen M, Kittisut N, et al. Predicting 10-year major adverse cardiac events using multi-source modalities with XGBoost. medRxiv 2025;August 29. CrossRef [Preprint]

35. Li ML, Shi RY, Zheng JY, et al. Myocardial MRI cine radiomics: a novel approach to risk-stratification for major adverse cardiovascular events in patients with ST-elevation myocardial infarction. J Magn Reson Imaging 2025;62:430–43. CrossRef

36. Li Z, Yang X, Zhang D, et al. Exploration of oral microbiota alteration and AI-driven non-invasive hyperspectral imaging for CAD prediction. BMC Cardiovasc Disord 2025;25:102. CrossRef

37. Pezel T, Toupin S, Bousson V, et al. A machine learning model using cardiac CT and MRI data predicts cardiovascular events in obstructive coronary artery disease. Radiology 2025;314:e233030. CrossRef

38. Zhang J, Xu J, Tu L, Jiang T, Wang Y, Xu J. A non-invasive prediction model for coronary artery stenosis severity based on multimodal data. Front Physiol 2025;16:1592593. CrossRef

39. Zou Q, Qiu T, Liang C, et al. Multimodal prediction of major adverse cardiovascular events in hypertensive patients with coronary artery disease: integrating pericoronary fat radiomics, CT-FFR, and clinicoradiological features. Radiol Med 2025;130:767–81. CrossRef
